# Explaining Gender Differences in Caries: A Multifactorial Approach to a Multifactorial Disease

**DOI:** 10.1155/2010/649643

**Published:** 2010-03-16

**Authors:** Maria Ferraro, Alexandre R. Vieira

**Affiliations:** Department of Oral Biology, School of Dental Medicine, University of Pittsburgh, Pittsburgh, PA 15261, USA

## Abstract

Many studies have demonstrated that caries rates are higher in women than in men. This review attempts to provide an explanation for this trend by examining each factor which contributes to caries and how the factor differs in men and women. Evidence has been provided to demonstrate that caries risk factors for women include a different salivary composition and flow rate, hormonal fluctuations, dietary habits, genetic variations, and particular social roles among their family. Systemic diseases that have been found to be associated with caries have also been found to have an association with the female gender. An extended exposure to the oral cavity or a more cariogenic oral microflora has not been proven to contribute to higher caries in women. Further research in these areas could be done in the future to explain their contribution, or lack thereof, to a higher caries rate in women.

## 1. Introduction

The significant impact of caries on the world's population makes the disease an important topic of understanding. The development of caries is multifactorial, depending on many interacting variables to promote its development. In particular, the presence of bacteria, a substrate for the bacteria (food/sugars), the host's oral environment, as well as the passing of time are the main contributing factors in the formation of caries. Epidemiological and clinical studies, through the use of tools such as DMFT and DMFS scores, have revealed a consistent trend in caries development, with females having higher prevalence than males [1]. The mechanisms underlying the reasoning for this trend can possibly be explained by an investigation of the suggested factors involved in caries development.

## 2. Genetic Contributions: *AMELX*

The underlying mechanisms of any genetic contributions to the increased prevalence of caries in females versus males can be speculated to reside in the sex chromosomes, exhibiting sex-linked modes of inheritance. Genes present on the X or Y chromosome whose function affects those factors which contribute to the development of caries can be investigated. Variations in these genes would alter the host's oral environment and the host's response to the initiation of caries.

The Amelogenin (AMELX) gene resides on the p arm of the X chromosome. Its locus is Xp22.31-p22.1 [2]. This gene and its protein product contribute to enamel formation in the dentition. The amelogenin protein constitutes 90% of the enamel matrix [3]. A mutation/deletion in the AMELX gene results in X-linked amelogenesis imperfecta [2]. There is a possibility that a deficient amelogenin gene or a decreased amount of amelogenin protein leads to disruption of formation of enamel matrix and therefore increased caries susceptibility [3]. 

The possibility of such a “caries susceptibility amelogenin variant” has been investigated. These studies explain that in females, it is possible for this kind of variation in AMELX to occur through the mechanisms of X inactivation and mosaicism. Normally, the inactivation of one X chromosome is random, with 1 : 1 distribution of the two AMELX genes inherited in females on the X chromosomes (mosaicism in regards to the X chromosome, since one comes from one parent and the other comes from the other parent). If a variant allele were present, the normal alleles that make up 50% of the genes could compensate and override any disease caused by the variant allele. However, if the X-inactivation is biased to favor those chromosomes with the deleterious variant AMELX allele, and the normal allele is nonrandomly inactivated, it would be expected that an increase in caries would be observed [3].

One study of 110 individuals used single-nucleotide polymorphism markers to genotype ameloblastin alleles. Individuals with “higher caries experience” (DMFT ≥3) were found to have an association with a variant allele marker for Amelogenin [3]. Another study using similar methods to target specific ameloblastin allele variants investigated the relationship in children. Overrepresentation of a particular allele marker (C allele) was seen in cases with DMFT scores higher than 8 [2]. These two studies have supported a link between the AMELX gene and caries rate. 

If an AMELX gene variant affects females who exhibit mosaicism for the X chromosome, it would be expected that this variant would also increase caries incidence in males since they too have only one copy of the gene on the X chromosome. To explain why this is not observed, the homologous AMELY gene on the Y chromosome (locus Yp11.2) has been suggested to be involved. A male with a normal and active AMELY gene may display compensation for a caries susceptibility AMELX allele on his X chromosome [3]. Another way to explain the role of AMELY in caries susceptibility is to consider its production of the amelogenin protein. AMELY gene only expresses 10% of amelogenin that is expressed by AMELX [2]. However, this additional 10% is not attained by females exhibiting X inactivation. Therefore, males may be expressing a greater amount of amelogenin, contributing to the strength of the tooth and less caries susceptibility of the host [2]. These proposed mechanisms of AMELY may be one way to explain why when exploring the role of amelogenin on caries formation, females exhibit greater prevalence than males.

## 3. Saliva

The composition and flow rate of saliva in the host oral environment seem to be another source of susceptibility of caries formation in women. Saliva plays a protective role in the oral cavity through its buffering, mechanical washing, antimicrobial, and remineralization activities. However, the flow rates of saliva and compositional analysis have been shown to be generally less protective in women than in men. Additionally, the hormonal fluctuations in women tend to play a role in the less protective composition and flow rate of saliva.

When the flow rates of resting whole saliva and stimulated parotid saliva in participants aged 20 and older were measured using timed expectoration and a Lashley cup respectively, differences were found between genders. In all age groups, females were found to have a lower mean flow rate of whole saliva than males, with significant differences in the 80+ age group (*P* < .02). In addition, the overall mean parotid salivary flow rate in females (0.45 mL/min) was significantly lower than the mean parotid salivary flow rate in males (0.59 mL/min *P* < .05) [4]. These findings have been supported in a more recent study on minor salivary gland secretion rates. By measuring the salivary rate from 142 individuals using The Periotron 6000 model 2, investigators found that the salivary flow rates from the buccal and labial glands in women were lower than those in men, especially in the elderly participants [5]. A lower salivary flow rate in females puts them at a higher risk for caries because they lack more of saliva's mechanical washing, buffering, and remineralization benefits.

In addition, the report by Ellaison et al. (2006) investigated the salivary IgA concentrations of the men and women participants. Salivary IgA is an immunoglobulin found in the oral cavity which is protective against caries. Results of this study show a difference in IgA concentration between men and women, with women having a lower concentration of the protective IgA from minor glands, (buccal, palatal, and labial) [5]. Information was obtained using enzyme-linked immunosorbent assay (ELISA) technique. Samples from the buccal glands showed mean IgA concentrations of 95.2 ± 76.9 *μ*/mL in women as compared to 155 ± 160 *μ*/mL in men, in the palatal glands, 121.3 ± 139.5 *μ*/mL in women and 210.4 ± 249.7 *μ*/mL in men, and in the labial glands, 46.2 ± 31.0 *μ*/mL in women and 58.8 ± 40.2 *μ*/mL in men [5]. It appears that males have inherently higher concentrations of IgA immunoglobulin to defend their oral surfaces against carious activity.

## 4. Pregnancy

A compelling reasoning why women have greater caries activity than men argues that pregnancies have several negative effects on the oral cavity environment. In general, the experience of pregnancy includes immune suppression, cravings, hormonal fluctuations, salivary alterations, and other physiological changes that would be expected to adversely affect the host resistance to caries.

Hormonal fluctuations of estrogen occur in females during pregnancy, menstruation, and puberty. These elevated estrogen levels can lead to significant changes in the environment of the oral cavity. Clinical studies have investigated perhaps the most severe of these hormonal fluctuation events, pregnancy. Lukacs and Largaespada (2006) discussed rat studies in which a causal link was found between caries rate and estrogen levels, but similar androgen level fluctuations experienced by males did not show this same kind of link or even a correlation [1]. 

Pregnancy can also have negative effects on salivary flow, impairing the protective washing and buffering mechanisms of saliva against caries development. The study discussed earlier by Eliasson et al. (2006) compared resting whole saliva rates of pregnant women to control (nonpregnant) women and found that the pregnant women had a mean secretion rate less than their control counterparts (0.21 ± 0.13 versus 0.30 ± 0.16 mL/min).

An anthropologic argument by Lukacs (2008) suggests that the development of agriculture may be indirectly responsible for an increased caries rate in females. Lukacs argues that the transition to agriculture is associated with an increase in fertility due to a more sedentary lifestyle and need for division of labor (as opposed to foraging and hunting). According to Lukacs, this would account for a decline in women's oral health related to the adverse effects of pregnancy on the oral environment [6].

## 5. Substrate/Diet

The presence of sucrose for *Streptococcus mutans'* metabolism is an additional factor in the establishment of a cariogenic environment. Dietary habits can have a major impact based on the form and frequency of the food. In many cultures historically, women have been the family member with the responsibility of food preparation. This would allow easier access to foods and snacks outside of mealtime, which provide bacteria in their oral flora with more substrate for caries development [7]. Particular dietary routines have been shown to increase the incidence of caries. In particular, vegetarian diets have been investigated in this aspect. Indian subjects are of particular interest in dietary habits because many have been vegetarian their whole life, offering a specific population. In a study of caries prevalence among 104 Indian participants ages of 5–72, patients with a vegetarian diet were found to have the highest numbers of caries when compared to those with a “vegetarian + tobacco” diet and a “nonvegetarian” diet. The investigators attribute this to a lack of putrefaction, a result of protein consumption, which contributes to the formation of a less acidic oral environment [8].

With the discovery that a vegetarian diet among Indian participants contributes to caries formation, it might be expected that more women are vegetarian than men, and this can be one way to explain their higher prevalence of caries. An epidemiological study by Shah (2003) included 1240 participants in the Southern Delhi area of India. Using survey techniques, investigators reported that more of the women participants were vegetarian than men (71% versus 56%) [9]. With the earlier described findings that vegetarian diets contribute to caries formation, this information can be added to the list of factors causing the gender disparity in caries prevalence.

## 6. Psychosocial and Economic Factors

Although clinical studies have been revealing in the reasons behind gender prevalence in females, epidemiological studies can also contribute to the cause. Women's role in society has been suggested as a causative factor in caries development. A study by Shah investigated sociodemographic gender differences in subjects and related them to health and disease. Shah's study included 1240 participants aged 60 and older in India and used survey techniques to evaluate the status of women in this region. In his findings, Shah includes a higher percentage of women being low SES (35% versus 2.8% in males), widowed (24.3% versus 13.9% in males), and economically dependent (79% versus 5% in males). Women also reported a lower level of literacy (35.5% versus 57.6% in males) and a lower sense of being loved and respected by their family (7% versus 92% in males) [9]. All of these factors contribute to a greater difficulty in an understanding of the disease process, ability to prevent the caries development process, and access to care for women.

Shah also includes information on the general population of women, pointing out that more women are single parents subject to the stress of care giving and also at an economic disadvantage. Women are more subject to domestic violence and eating disorders. They also live longer, increasing their prevalence of systemic issues and their use of medications. All of these factors can have an effect on host defense responses which would fight disease in the oral cavity.

## 7. Time

In the pattern of tooth eruption, females tend to acquire their teeth at an earlier age than males. A female's teeth are therefore exposed to the oral environment, bacteria, and bacterial substrates for a longer time than the teeth of a male the same age, providing more opportunity for the caries developing process to take place. Because of this trend, one might expect that the gender disparity in caries prevalence would be evident at an early age, but some recent investigations have found contradicting support of this expectation.

When investigating the prevalence and causes of Nursing Caries, it was discovered that of 544 children, the prevalence of Nursing Caries was greater in girls than in boys, with 23.5% prevalence in girls compared to 16.5% prevalence in boys. The children included in this study were 18 to 60 months old [10]. This study coincides with the idea that girls should be demonstrating more caries than boys early in life. However, Nursing Caries is a specific form of carious disease. Data from this investigation only reports the presence or absence of Nursing Caries, and does not report the gender comparative results in the form of DMFT or other more complete caries experience indicator. 

An examination of 771 children who were 2 years old in Zurich identified the number of initial or cavitated lesions in children and reports male gender as a risk factor for caries [11]. However, a study of prevalence of dental health problems in children of Kerala examined 1068 children from ages 12–15 years old. They reported boys and girls being almost equally affected by caries, or with females slightly more affected (49% male versus 51% female) [12].

In a cross sectional examination of 10 and 11 years olds in southern Italy, a significant difference was found between the young boys and girls. The mean DFT for boys was 3.20 versus a mean DFT of 1.96 in girls. This difference was statistically significant, in finding that in this group, it was more common for the male children to have caries than the female children [13]. In India a similar investigation was done which demonstrates a window of time where prevalence switches from male to female. The study population was limited to 5 and 12 year old children (1009, 5-year-olds and 1013, 12 year olds). In the 5 year old age group, 47.4% of those children with caries were male, while 41.1% of those children were female. However, in the 12-year-old age group a greater percentage of those children with caries were female (24.1% female versus 20.6% male). Implications made from this study can suggest that there is a time between 5 and 12 years old when higher caries prevalence in children switches from male to female [14]. 

Although, intuitively, the earlier exposure of female teeth to the oral cavity should provide explanation for the higher incidence of caries in females, contradictory information has been found to support this idea in children.

## 8. Bacteria

The primary initiator of dental caries is *Streptococcus mutans.* The presence of *S. mutans* can greatly increase the risk of caries if the host's defense mechanisms do not override the bacteria. It can be proposed that females are found with more caries because they harbor more of the caries causing bacteria (i.e., *S. mutans*). A study of twins (both monozygotic and dizygotic) tested the amount of *S. mutans* in participants using the Stripmutans test. Results of this study found no statistically significant gender differences in the amount of *S. mutans*. These results included those from 28 pairs of dizygotic opposite gender twins [15]. According to this study, it appears that there is no gender bias in the amount of *S. mutans* of the individual to contribute to caries formation. Additionally, an investigation by Loyola-Rodriguez et al. found no statistically significant differences in *S. mutans or S. sobrinus* (another caries causing microbe) between genders after inoculating saliva of caries-free and caries active children [16].

## 9. Systemic Correlations

The investigation in gender preference in caries rates can look at correlations with systemic diseases. If we know that there is a positive correlation between the female gender and caries rates and if positive correlations are found between caries and systemic disease, it could be expected that more women would be seen with the same systemic disease. This expectation was found to be true in a recent study aiming to link systemic diseases and oral conditions. With the use of the Dental Registry and DNA Repository at the University of Pittsburgh School of Dental Medicine, the medical history and DMFT/DMFS scores of 318 subjects were examined. An association was found between asthma and those individuals with DMFT above 15 and DMFS above 50. A similar association was found between high DMFT/DMFS scores and epilepsy [17]. According to our expectations, we would therefore anticipate that more women than men would report having asthma or epilepsy. Upon further inspection of those with these systemic diseases, another association was found in the prevalence of asthma, with females experiencing a higher occurrence (*P* = .04) [17].

## 10. Conclusions

In explaining the consistent trend of caries rates being higher in females than males, all contributing factors must be considered. There is no one reason for this disparity. Women's roles in their community (caretaker, meal preparation, etc.), along with other social factors, such as differing salivary flow rates and compositions, dietary habits, hormonal changes during pregnancy, and particular variants of the *AMELX* gene must all be included in the assessment of an individual woman's caries risk assessment. Substantial evidence has not been found concerning the contributions of the longer time female teeth are exposed to the oral cavity or concerning a differing microbial oral flora which might enhance the caries developing process. More research is needed to define the role of these two possible contributors more clearly in order for us to more completely understand the development of caries in women and to anticipate the disease process before it begins.
